# Active MMP-8 as a Biomarker of Peri-implant Health or Disease

**DOI:** 10.1055/s-0042-1753454

**Published:** 2022-09-05

**Authors:** Vithleem Xanthopoulou, Ismo Räisänen, Timo Sorsa, Dimitra Sakellari

**Affiliations:** 1 Department of Preventive Dentistry, Periodontology and Implant Biology, Dental School, Aristotle University of Thessaloniki, Thessaloniki, Greece; 2 Department of Oral and Maxillofacial Diseases, Head and Neck Center, University of Helsinki and Helsinki University Hospital, Helsinki, Finland; 3 Division of Periodontology, Department of Dental Medicine, Karolinska Institutet, Stockholm, Sweden

**Keywords:** aMMP-8, peri-implant health, peri-implant disease

## Abstract

**Objectives** This study investigated the potential of testing for active matrix metalloproteinase-8 (aMMP-8) by a quantitative point-of-care (PoC), chairside, lateral flow immunotest as a biomarker for the presence or absence of peri-implant diseases.

**Materials and Methods** Eighty healthy patients with implants were recruited. The samples were collected from peri-implant sulcular fluid and quantitatively analyzed for aMMP-8. Clinical indices, which included probing depth, clinical attachment loss, bleeding on probing, and plaque, were recorded and radiographic assessments were performed.

**Statistical Analysis** Comparisons of aMMP-8 levels and clinical parameters were analyzed by the Kruskal–Wallis test and the pairwise post hoc Dunn–Bonferroni test. A receiver operating curve analysis was used to analyze the diagnostic ability of aMMP-8 and the correlation between aMMP-8 and probing depth of the sampled site was sought by Spearman's rho and the coefficient of determination ( *R* ^2^ ).

**Results** Statistical analysis revealed statistically significant differences of aMMP-8 levels between the healthy and the mucositis and peri-implantitis groups, and between the mucositis and the peri-implantitis groups. Increasing probing depths of the sampled site and aMMP-8 levels were significantly correlated.

**Conclusions** These data suggest that the aMMP-8 PoC test can be a beneficial adjunctive tool for early identification and screening of the risk of peri-implant diseases and progression.

## Introduction

Osseointegrated dental implants are currently widely used by dentists to achieve the oral rehabilitation of patients. Albeit the widespread popularity among clinicians and patients, complications are not rare, the most significant being peri-implantitis.

According to the most recent classification of periodontal and peri-implant diseases, 1 peri-implantitis is defined as the pathological condition around dental implants characterized by inflammation in the peri-implant mucosa and progressive bone loss. In contrast, peri-implant mucositis is characterized by inflammation in the implant surrounding mucosa without concomitant bone loss. 2 The prevalence of peri-implantitis according to a recent meta-analysis is estimated to be 12.8% at the implant level and 18.5% at the patient level, although most studies reveal a wide reported range. 3 Thus, the global burden of peri-implantitis and related cost of therapy are difficult to precisely evaluate.

Diagnosis of peri-implant disease is currently based on clinical and radiographic findings, but these assessments evaluate already established disease and do not offer the possibility of early detection and treatment of pathological conditions. 4 5 Therefore, the incorporation of a biomarker to the diagnostic procedure would be very useful, especially with the anticipated increase in the treatment demand with implants, until 2030. 6

It is known in the literature that active-matrix metalloproteinase-8 (aMMP-8) has been investigated as a biomarker of peri-implant tissue breakdown, based on the fact that an increase of the levels of the activated enzyme has been consistently observed in pathological peri-implant conditions. 7 8 9 10 11 12 13 14 15 16

The aim of the present study was to investigate the potential of testing for aMMP-8 by a quantitative point-of-care (PoC), chairside lateral-flow immunotest as a biomarker for the presence or absence of peri-implant diseases.

## Materials and Methods

The study was designed as a cross-sectional study. Eighty consecutive patients (42 males and 48 females; mean age: 56.66 + 11.27 years) were recruited from the Department of Periodontology and Implant Biology of the Dental Faculty, School of Health Sciences, Aristotle University of Thessaloniki, Greece, from September 2021 to September 2022. Criteria for inclusion were the absence of systemic diseases, infectious diseases (human immunodeficiency virus/hepatitis B virus/hepatitis C virus infection), periodontal treatment or use of antibiotics for the last 6 months, and the presence of an implant with a functional load for at least 1 year. Patients with diabetes or other immunomodulating diseases were also excluded from the study. One implant per patient was included in the study. In case more than one implant was present in the dentition, there was a random selection of the implant to be included in the study. Participants signed an informed consent form and the study was approved by the Ethical Committee of the School of Dentistry, Aristotle University of Thessaloniki (#10/26.02.2020).

Before clinical examination and diagnosis, a sample was taken from the peri- implant sulcular fluid (PISF) for analysis of aMMP-8 levels preferably from either the mesio-buccal or the disto-buccal site of the implant. Specifically, after isolating the area with cotton swabs to avoid saliva contamination and removal of supragingival plaque, the implant was air dried and fluid from PISF was collected with paper strips (Periopaper), which were inserted into peri-implant sulcus (1–2 mm subgingivally) for 30 seconds. Strips that were visually contaminated with blood or saliva were discarded. The collected PISF was analyzed for quantitative assessment of aMMP8 levels using the Implantsafe test (Dentognostics GmbH) and the accompanying digital reader (ORALyzer®), according with the manufacturer's instructions, and levels were expressed in ng/mL.

Following collection and analysis of PISF, clinical examination was performed, which included the following measurements: full-mouth plaque score, percentage of sites positive on bleeding on probing (BOP), probing depth (PD), and clinical attachment level (CAL) of the implant. All measurements were recorded at six sites per implant with a 15-mm scale periodontal probe and graded per 1 mm (Hu-Friedy® CP-12, #30). All assessments were performed by the same calibrated examiner (V.X.).

X-ray imaging of the implants followed the clinical examination. The X-ray was taken using a digital X-ray imaging system with phosphor plates (SCANORA 37, Software SOREDEX). For each case, all X-rays were taken using the same irradiation time which corresponds to the time indicated on the X-ray apparatus depending on the dental group to which the implant belongs. The type of implants and their prosthetic restoration were also evaluated.

Following clinical and radiographic examination, a diagnosis was made regarding the periodontal status of the whole dentition and the implant under investigation, according to the 2018 Classification of Periodontal and Peri-implant Diseases. 1 2

## Statistical Analysis

Comparisons of aMMP-8 levels and clinical parameters were analyzed by the Kruskal–Wallis test and pairwise post hoc Dunn–Bonferroni test. A receiver operating curve (ROC) analysis was used to analyze the diagnostic ability of aMMP-8 (ng/mL) and the area under the curve (AUC) was calculated and the correlation between aMMP-8 levels and PD of the sampled site was sought by the Spearman's rho and the coefficient of determination ( *R* ^2^ ). Differences of distribution of age and sex among groups were sought by applying the z-test for column proportion with Bonferroni correction. All tests were set at the 0.05 level of significance.

## Results

Patients with healthy implants, peri-implant mucositis, or peri-implantitis did not differ regarding age or sex distribution (age range: 52.5 + 12.9, 57.6 + 10, and 62.6 + 7 years, respectively, z-test for column proportion with Bonferroni correction, *p* > 0.05). Regarding PD of the investigated implant, statistically significant differences were observed, between healthy implants and peri-implantitis (3.11 ± 0.43 and 5.87 ± 1.90 mm, respectively) and between implants with peri-implant mucositis and those with peri-implantitis (3.21 ± 0.31 and 5.87 ± 1.90 mm, respectively) (Kruskal–Wallis test, *p* < 0.05). The same pattern of statistically significant differences was observed for CAL assessments with no differences observed between healthy implants and peri-implant mucositis (3.29 ± 0.60 and 3.28 ± 0.32 mm, respectively) (Kruskal–Wallis test, *p* > 0.05) but statistically significant differences observed between healthy implants and peri-implantitis (3.29 ± 0.60 and 6.19 ± 1.98 mm, respectively) as well as between peri-implant mucositis and peri-implantitis (3.28 ± 0.32 and 6.19 ± 1.98 mm, respectively) (Kruskal–Wallis test, *p* < 0.05). Regarding BOP, only healthy implants displayed statistically significant differences versus implants with peri-implant mucositis or peri-implantitis (no bleeding vs. 0.65 ± 0.20% and 0.88 ± 1.64%, respectively) (Kruskal–Wallis test, *p* < 0.05).

**Fig. 1:**
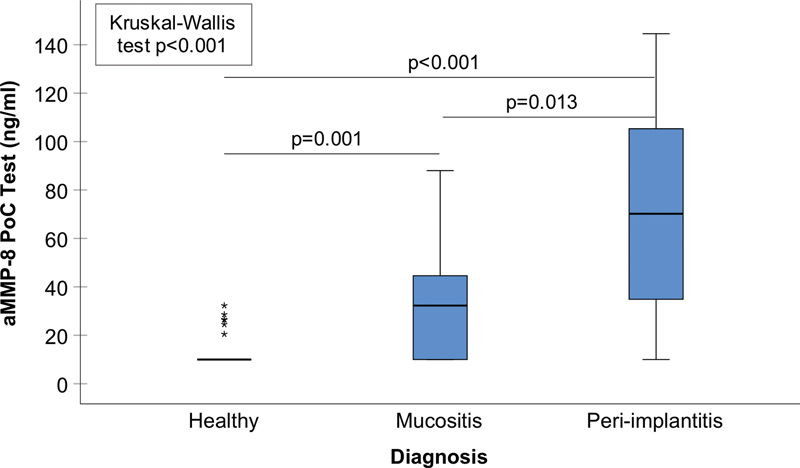
A boxplot of the aMMP-8 levels (ng/mL) measured by the aMMP-8 PoC test for healthy ( *N* = 27), mucositis ( *N* = 41), and peri-implantitis ( *N* = 12) groups. The omnibus analysis of these three groups was conducted with the Kruskal–Wallis test and pairwise post hoc analysis with Dunn–Bonferroni post hoc method. Statistically significant differences between the healthy and the mucositis ( *p* = 0.001), the healthy and the peri-implantitis ( *p* < 0.001) groups, as well as the mucositis and the peri-implantitis groups ( *p* = 0.0013) were observed.

**Fig. 2:**
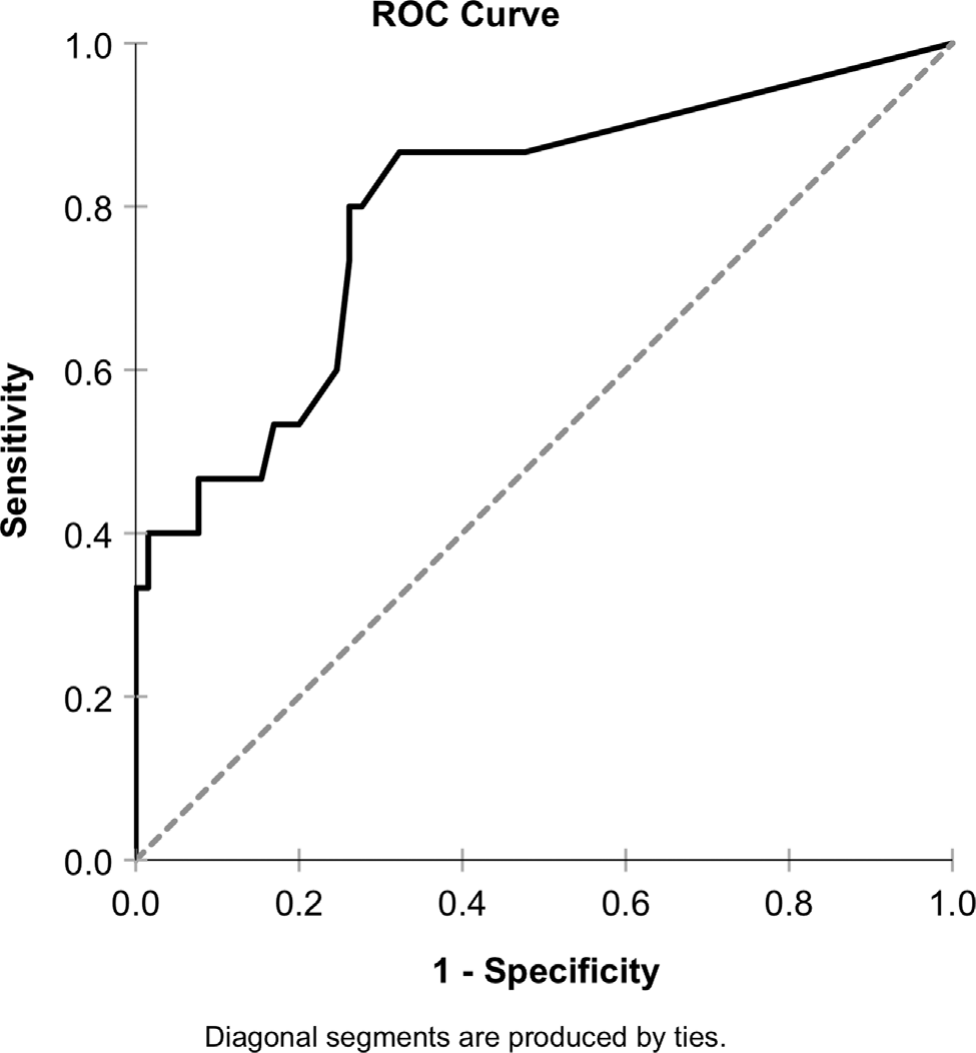
Receiver operating characteristic curve analysis of aMMP-8 ability to discriminate implants with at least one site with probing depth ≥5 mm together with at least one site with bleeding on probing (AUC = 0.798). Optimal cutoffs were calculated by Youden's index (aMMP-8: 32.15 ng/mL, sensitivity: 0.867, specificity: 0.677). AUC, area under the curve.

**Fig. 3:**
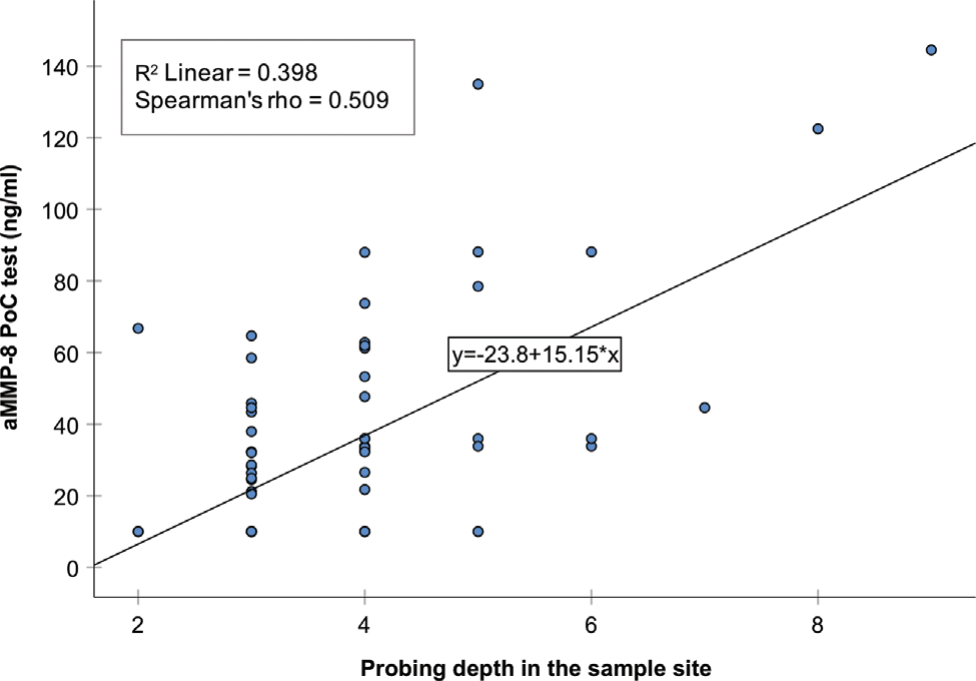
Correlation between probing depth of the sampled site of all implants and aMMP-8 levels (ng/mL). A statistically significant positive correlation was observed between aMMP-8 levels and increasing probing depths (PDs) (rho = 0.509, *R* ^2^ = 0.398; *p* < 0.001).

A boxplot of aMMP-8 (ng/mL) for the three categories of peri-implant conditions and their statistical comparisons is depicted in Fig. 1 . Statistical comparisons were conducted with the Kruskal–Wallis test and pairwise post hoc analysis with the Dunn–Bonferroni post hoc method at the 0.05 level, which revealed statistically significant differences between the healthy and the mucositis and peri-implantitis groups, as well as the mucositis and the peri-implantitis groups. A ROC analysis was also performed to analyze the diagnostic ability of aMMP-8 (ng/mL) to discriminate patients with at least one site with ≥5 mm PD together with at least one site with BOP ( Fig. 2 ). AUC was also calculated (AUC = 0.798; 95% confidence interval: 0.665–0.932; *p* < 0.001), and optimal cutoff a-MMP8 value was estimated by Youden's index (aMMP-8: 32.15 ng/mL; sensitivity: 0.867; specificity: 0.677). The correlation between PDs of the sampled site of all implants was calculated by both Spearman's rho and the coefficient of determination (R ^2^ ) using the fitted linear regression model. Both analyses displayed a statistically significant positive correlation between increasing PDs and aMMP-8 levels (rho = 0.509, *R* ^2^ = 0.398; *p* < 0.001) ( Fig. 3 ).

## Discussion

In the present study, the diagnostic potential of an aMMP-8 PoC test to discriminate peri-implant conditions was investigated. According to the depicted data, it was observed that increased levels of aMMP-8 in PISF was significantly higher in implants with peri-implantitis when compared with healthy implants or implants with mucositis, suggesting destruction of peri-implant tissues by collagenolysis. In addition, ROC analysis revealed a high diagnostic potential of the aMMP-8 test to discriminate patients with least one site with ≥5 mm PD together with at least one site with BOP on the implant, before the clinical examination.

These data suggest that an aMMP-8 PoC test can be a beneficial adjunctive tool for early identification and screening for peri-implant diseases.

Moreover, statistical analysis displayed a statistically significant correlation between the increasing levels of aMMP-8 in PISF and increasing PD in the sampled site. These findings are in agreement with previous studies that have shown a direct correlation between active MMP-8 levels and periodontal/peri-implant clinical parameters, 10 13 15 17 18 while this strong correlation was not observed between the levels of the total enzyme (both active and latent forms). 19

As discussed earlier, to maintain periodontal and peri-implant health, it is crucial to establish collagen balance. The presence of collagen degradation is not feasible to be detected with clinical examination and radiographs, which are the regular assessments of the peri-implant tissues at the dental settings. However, analysis of active MMP-8 in oral fluids is a noninvasive method to recognize the process of active collagenolysis. Based on the results of this analysis, the actual state or disease activity of periodontal/peri-implant tissues can be assessed, as suggested by the present and recent studies and therefore, clinicians can alert patients to improve their compliance in strict recall programs and in oral hygiene procedures, as well as provide them prevention with protocols to avoid further periodontal and peri-implant tissue breakdown. 18 20 Previous studies have used various definitions of peri-implant disease and therefore, results are not easy to compare. In the present investigation, the criteria, and definitions of the 2018 classification of peri-implant conditions have been applied but despite the promising findings, the confined number of participants can be considered a limitation. Further studies with larger patient samples are required to consistently establish the value of this chairside test for identification of peri-implant tissue destruction.

## Conclusion

Taken together, it is suggested from the present findings that the aMMP-8 enzyme chairside lateral flow immunotest can be a beneficial adjunctive diagnostic tool for early identification and screening of the risk of peri-implant diseases.

Future well-designed studies are required to confirm these findings and also investigate the possible utility of these tests for identifying disease progression, as well as the success and failure of various treatment modalities during maintenance.
